# Isolation of two strains of West Nile virus during an outbreak in southern Russia, 1999.

**DOI:** 10.3201/eid0604.000408

**Published:** 2000

**Authors:** D K Lvov, A M Butenko, V L Gromashevsky, V P Larichev, S Y Gaidamovich, O I Vyshemirsky, A N Zhukov, V V Lazorenko, V N Salko, A I Kovtunov, K M Galimzyanov, A E Platonov, T N Morozova, N V Khutoretskaya, E O Shishkina, T M Skvortsova

**Affiliations:** D.I.Ivanovsky Institute of Virology, Moscow.

## Abstract

From July to September 1999, a widespread outbreak of meningoencephalitis associated with West Nile virus (Flavivirus, Flaviviridae) occurred in southern Russia, with hundreds of cases and dozens of deaths. Two strains of West Nile virus isolated from patient serum and brain-tissue samples reacted in hemagglutination-inhibition and neutralization tests with patients' convalescent-phase sera and immune ascites fluid from other strains of West Nile virus.


### Isolation of Two Strains of West Nile Virus during an Outbreak in Southern Russia, 1999

**D.K. Lvov,* A.M. Butenko,* V.L. Gromashevsky,* V.Ph. Larichev,* S.Ya. Gaidamovich,* O.I. Vyshemirsky,* A.N. Zhukov,† V.V. Lazorenko,† V.N. Salko,‡ A.I. Kovtunov,‡ Kh.M. Galimzyanov,§ A.E. Platonov,¶ T.N. Morozova,* N.V. Khutoretskaya,* E.O. Shishkina,* T.M. Skvortsova***

*D.I.Ivanovsky Institute of Virology, Moscow; †Center of State Sanitary-Epidemiological Inspection, Volgograd; ‡Center of State Sanitary-Epidemiological Inspection, Astrakhan; §Medical Academy, Astrakhan; ¶Central Institute of Epidemiology, Moscow

From July to September 1999, a widespread outbreak of meningoencephalitis associated with West Nile virus (Flavivirus, Flaviviridae) occurred in southern Russia, with hundreds of cases and dozens of deaths. Two strains of West Nile virus isolated from patient serum and brain-tissue samples reacted in hemagglutination-inhibition and neutralization tests with patients' convalescent-phase sera and immune ascites fluid from other strains of West Nile virus.

From July to September 1999, a widespread outbreak of meningoencephalitis occurred in southern Russia (Volgograd, Astrakhan, and Krasnodar regions). Approximately 1,000 cases with at least 40 deaths were reported. Natural foci of arbovirus infections have been reported in southern Russia (1-5). Clinical and epidemiologic investigations indicated that this outbreak could be associated with West Nile virus; preliminary serologic testing of patient samples confirmed the presence of the virus (6). We report further virologic testing of patient isolates from this outbreak.

### The Study

For virus isolation, we tested serum samples from 25 patients on days 4 to 6 of febrile illness, 18 samples of cerebrospinal fluid, and brain tissue samples taken from 5 patients at autopsy. The tissue and cerebrospinal fluid were analyzed for evidence of West Nile virus genome by reverse transcription-polymerase chain reaction (RT-PCR) primers on the basis of published NS5 and E genes (7,8). Virus was isolated by infection of 3- to 4-day-old suckling mice. Mice were injected intracranially with 0.01 mL of patient tissue, and blind passages were made on days 6 to 7 after inoculation. The suspension of brain tissue from previously injected, asymptomatic mice was inoculated intracranially into new mice. When mice began to show signs of illness, the brain tissue was examined for West Nile virus by hemagglutination (HT) and hemagglutination inhibition tests (HIT). A 10% suspension was prepared in 0.15 M NaCl and diluted fivefold with borate buffer solution to suppress nonspecific inhibitors. The suspension was then titrated at pH 6.4 with goose erythrocytes (9). Identification of the virus antigen in brain suspension of infected mice was also done by enzyme-linked immunosorbent assay (ELISA) with the direct sandwich method (9).

Immune ascitic fluids (IAF) of mice and convalescent-phase sera of patients in the current outbreak were used for identification of these strains and viruses by HIT, neutralization, and ELISA testing. Neutralization testing (NT) was done by the micro method in pig kidney cells with a single dilution of IAF in 10-fold dilutions of virus (Table 1). The results were assessed

Address for correspondence: D.K. Lvov, D.I. Ivanovsky Institute of Virology, Gamaleya Str., 16 Moscow, Russia, 123098; fax: 7-(095)-190-28-67.

Table 1. Neutralization index of strain LEIV 27889 Vlg in neutralization test with immune ascitic fluid

| Strain | Neutralization index |
|---|---|
| WNF 2266Ig (India) | 6.5 |
| WNF 22886G (India) | 8.5 |
| WNF Eg 101 | 8.5 |
| Kokobera | 6.0 |
| Karshi | 1.0 |
| Apoi | 3.0 |
| Usutu | 3.0 |
| JE | 3.5 |
| Tyuleniy | 2.0 |
| St. Louis | 0.5 |
| TBE | 0.5 |

according to a neutralization index calculated by the Reed and Mench method (9).

We examined brain tissue from five patients (63, 67, 71, 72, and 16 years of age) in the Volgograd region who died of meningoencephalitis. Flaviviruses and West Nile virus RNA were detected in all five samples by RT-PCR; however, virus was isolated only from the 16-year-old patient. In this case, suckling mice injected with brain tissue became ill on days 4 to 6. This incubation period decreased to 3 to 5 days on the second passage and to 3 days after subsequent passages. On the second passage, we detected hemagglutinins in mouse brain suspension of this virus at a titer of 1:128 at pH 6.4. The HIT for this isolate was inhibited by IAF to West Nile virus. Antigen of West Nile virus was also identified from mouse brain suspension by ELISA at titers of 1:80 to 1:160. This isolate was designated LEIV 22889 Vlg. All 18 samples of cerebrospinal fluid from patients were negative by RT-PCR and virus isolation in suckling mice.

Serum samples from 25 patients from the Astrakhan region were tested for virus isolation. Virus strain AST 986 was isolated in serum of one patient on days 7 to 8 after inoculation into suckling mice. The incubation period after the third passage was reduced to 3 days. Hemagglutinating antigen was identified in brain suspension of the mice on the second passage at titer 1:640, reciprocally with IAF of West Nile virus (Table 2).

Both strains LEIV 27889 Vlg and Ast 986 were reactive in HIT (Table 2). Antigens LEIV 27889 Vlg and strain LEIV Az-1640 of West Nile virus reacted in similar titers with IAF of all flaviviruses studied except yellow fever. When the strain LEIV 27889 Vlg was tested by NT (Table 1), virus was neutralized with IAF to all strains of West Nile and Kokobera viruses (Index of Neutralization 6.0-8.5). The identification of strains LEIV 27889 Vlg and Ast 986 was confirmed by HIT with convalescent-phase sera.

Table 2. Identification of the strains LEIV 27889 Vlg and Ast 986 by hemagglutination inhibition test with immune ascitic fluid and antigens of flaviviruses

| | Viral antigens[a] | | | | | | | |
|---|---|---|---|---|---|---|---|---|
| | 27889 | AST | LEIV | LEIV | LEIV | | YF | St. |
| IAF of viruses | Vlg | 986 | Az-1640 | Az-72 | Az-1628 | JE | (Dakar) | Louis |
| LEIV Az-1640 | 320[b] | nt | 1280 | 160 | nt | nt | nt | nt |
| LEIV Az-72 | 80 | nt | 160 | 160 | nt | nt | nt | nt |
| LEIV Az-1628 | nt | 640 | nt | nt | 1280 | nt | 640 | nt |
| Japanese encephalitis | 160 | 320 | 320 | 160 | 160 | 640 | nt | nt |
| Kokobera | 160 | nt | 320 | 160 | nt | nt | nt | nt |
| St. Louis | 160 | 160 | 320 | 160 | 80 | 0 | 0 | 160 |
| Usutu | 160 | nt | 160 | 320 | nt | nt | nt | nt |
| Apoi | 320 | nt | 320 | 160 | nt | nt | nt | nt |
| Karshi | 8 | nt | 80 | 80 | nt | nt | nt | nt |
| Tyuleniy | 160 | nt | 320 | 160 | nt | nt | nt | nt |
| Kama | 160 | nt | 160 | 160 | nt | nt | nt | nt |
| TBE | 20 | nt | 20 | 20 | nt | nt | nt | nt |
| Yellow Fever (Dakar) | 0 | nt | nt | nt | 0 | nt | 640 | nt |

nt = not tested; IAF = immune ascitic fluid.

[a]Isolates were identified by comparative testing with the following strains of West Nile virus: LEIV Az1640, Azerbaijan, 1967, from *Sitta europea* birds; LEIV Az1628, Azerbaijan, 1967, from *Turdus merula* birds; LEIV Az72, Azerbaijan, 1970, from *Ornithodorus capensis* ticks; 2269 Ig, Madras, 1956, from *Culex vishnui* mosquitoes; in Eg 101, 1951, from the serum of an Egyptian pediatric patient; and other flaviviruses: Japanese encephalitis (JE), St. Louis encephalitis (SLE), Yellow fever-Dakar (YF), tick-borne encephalitis (TBE), Kokobera (KOK), Usutu (USU), Apoi, Karshi (KSI), Kama, and Tyuleniy (TYU).

[b]quantity inverse IAF dilution.

### Conclusions

According to virologic and serologic data from the Center of Ecology of Viruses, D.I. Ivanovsky Institute of Virology, and collaborating laboratories, the West Nile virus-endemic area in the former Soviet Union includes Moldavia, Ukraine, Bielorussia, the southern area of European Russia (regions of desert, steppe, and deciduous forests) and western Siberia-Altai territory (steppe and combined forest-steppe), Armenia, Azerbaijan, Kazakhstan, Tajikistan, Uzbekistan, and Turkmenia. For the last 20 years, illness has been observed in Kazakhstan and the republics of Central Asia, Astrakhan region (in Russia), Ukraine, and Azerbaijan (1). High risk for exposure to West Nile virus has been observed in the desert territories of the Volga basin, especially in the river valleys, where an outbreak occurred in 1999.

The ornithophilic mosquito species *Culex modestus* is of great importance for circulation of West Nile virus in natural foci of bird colonies in the Volga Delta and in populated areas. Both *Culex p. pipiens* and *C. p. molestus* feed on wild, sylvan, and domestic birds, as well as humans. In the Volga Delta, 56 species of birds are involved in virus circulation. In the coastal area of the delta, the most important hosts are shore birds, especially the Gressores order: the green heron (*Nicticorax nicticorax,* 45% of which had antibodies), great cormorant (*Phalacrocorax carbo*), coot (*Fulica atra*), waterhen (*Gallinula chloropus*), and great grebe (*Podiceps cristatus*), and to a lesser extent gulls and terns (10). In the agricultural region of the Volga Delta, 20 species of birds (particularly rooks, crows, and pigeons) are involved in virus circulation (11). Less virus circulation is seen in other areas of the delta, in semidesert region of Astrakhan, and Kalmykia. In the Kuban and Terek River deltas, the most important birds are herons, coots, and some species of ducks.

In light of these data, the occurrence of West Nile virus outbreaks is not surprising. However, the high death rates and a wide range of infected populations are unusual and probably result from factors such as high temperature, extended breeding places of mosquitoes, migration of some groups of populations, and perhaps change in the virus genotype. The ecology of West Nile virus in southern Russia is similar to that in northeastern Romania, in the Danube Delta (12,13). A different ecologic situation was observed during the West Nile outbreak in New York in 1999 (8,14-17). The virus may have been introduced to the American continent by infected mosquitoes (eggs or larvae) from disease-endemic areas in Africa, Asia, or Europe by ships or airplanes. The urban subspecies *C. p. molestus*, which can reproduce without bloodsucking, may have introduced the virus; these ornithophilic mosquitoes become infected when they bite infected birds. The high susceptibility of these mosquitoes to Karshi virus, which closely resembles West Nile virus, was confirmed experimentally (18). The results of genome sequencing of strains isolated in the last epidemic and other West Nile strains previously isolated in the former Soviet Union will be described in the future.

Dr. Lvov is director of the D.I. Ivanovsky Institute of Virology, Russian Academy of Medical Sciences. His scientific interests focus on the ecology and epidemiology of arboviruses, viral hepatitis, and influenza.

### References

1. Lvov DK, Klimenko SM, Gaidamovich SY. [Arboviruses and arboviral infections.] Moscow: Meditsina; 1989. p. 1-333. (In Russian)
2. Lvov DK. Ecological soundings of the former USSR territory for natural foci of arboviruses. In: Lvov DK, editor. Arboviruses. Sov Med Rev E: Virology. New York: Harwood Inc.; 1993. p. 1-47.
3. Lvov DK. Arboviral zoonoses of Northern Eurasia (Eastern Europe and the Commonwealth of Independent States). In: Beran GW, Steel JH, editors. Handbook of zoonoses. 2nd ed. Boca Raton: CRC Press; 1994. p.237-60.
4. Lvov DK, Deryabin PG, Myasnenko AM, Skvortsova TM, Aristova VA, Butenko AM, et al. Atlas of natural foci of virus infections in the Russian Federation, Federal Department of Medico-Biological and External Problems at Ministry of Public Health of RF. Moscow: D.I. Ivanovsky Institute of Virology RAMS; 1995, p. 1-187.
5. Lvov DK. West Nile Fever (Survey). Voprosi of Virology 2000;45:4-9. (In Russian).
6. Lvov DK, Butenko AM, Gaidamovich SY, Larichev VPh, Leschinskaya EV, Lozarenko VV, et al. Epidemic outbreak of meningitis and meningoencephalitis in Krasnodar territory and Volgograd region provoked by West Nile fever virus. Vopr. Virusol 2000;45:37-8. (In Russian).
7. Savage HM, Ceianu C, Nicolescu G, Karabatsos N, Lanciotti R, Vladimirescu A, et al. Entomologic and avian investigations of an epidemic of West Nile fever in Romania in 1996 with serologic and molecular characterization of a virus isolate from mosquitoes. Am J Trop Med Hyg 1999;61:600-11.

8. Briese T, Jia XY, Huang C, Grud LJ, Lipkin WI. Identification of Kunjin West Nile-like flavivirus in brains of patients with New York encephalitis. Lancet 1999;354: 1261-2.
9. Clarke DH, Casals J. Techniques for haemagglutination and haemagglutination-inhibition with arthropod-borne viruses. Am J Trop Med Hyg 1958;7:561-73.
10. Berezin VV, Semenov BF, Reschetnikov IA, Baschkirtsev VN. Importance of birds in the natural cycle of arboviruses transmitted by mosquitoes in the Volga delta. In: Transcontinental connections of migrating birds and their role in arbovirus distribution. Novosibirsk: Nauka; 1972. p.310-3. (In Russian).
11. Butenko AM, Chumakov MP, Bashkirtsrev VN. Isolation of West Nile virus in the Astrakhan region from mosquitoes and crows. In: Aetiology, epidemiology and clinics of CHF and West Nile fever, Astrakhan. Astrakhan: Institute of Poliomyecitis and Viral Encephalitis, Astrakhan Sanitary-Epidemiological Station;1969. p.39-40. (In Russian).
12. Yarovoj PI. Antibodies to some flaviviruses in sera of habitants of north-Moldavian forest-steppe landscape. In: Gaidamovich SY, editor. Arboviruses. Moscow: D.I. Ivanovsky Institute of Virology; 1981. p.120-1. (In Russian).
13. Vinograd IA, Beletskaya GV, Chumachenko SS, Omelchenko GA, Lozinsky IN, Yartus DS, et al. Ecological aspects of arboviruses in the Ukraine SSR. In: Vinograd IA, Beletskaya GV, Chumachenko SS, Omelchenko GA, Lozinsky IN, Yartus DS, et al. Ecology of viruses and foci of arbovirus infections. Moscow: D.I. Ivanovsky Institute of Virology; 1989. p. 21-7. (In Russian).
14. Anderson JF, Andreadis TY, Voshbrinck CR, Tirrell S, Wakem EM, French RA, et al. Isolation of West Nile virus from mosquitoes, crows and a Cooper's hawk in Connecticut. Science 1999;286:2331-3.
15. Lanciotti RS, Roehrig JT, Deubel V, Smith J, Parker M, Steele K, et al. Origin of the West Nile virus responsible for an outbreak of encephalitis in the Northern United States. Science 1999;286:2333-7.
16. Centers for Disease Control and Prevention. Update: West Nile virus encephalitis—New York, 1999. MMWR Morb Mortal Wkly Rep 1999;48:890-2.
17. Centers for Disease Control and Prevention. Update: West Nile virus encephalitis—New York, 1999. MMWR Morb Mortal Wkly Rep 1999;48:944-55.
18. Khutoretskaya NV, Aristova VA, Rogovaya SG, Lvov DK, Karimov SK, Skvortsova TM. Experimental study of reproduction of Karshi virus (Flaviviridae, Flavivirus) in some species of mosquitoes and ticks. Acta Virol 1985;29:231-6.
